# Ultrafast syn-eruptive degassing and ascent trigger high-energy basic eruptions

**DOI:** 10.1038/s41598-017-18580-8

**Published:** 2018-01-09

**Authors:** Marisa Giuffrida, Marco Viccaro, Luisa Ottolini

**Affiliations:** 1 Università di Catania, Dipartimento di Scienze Biologiche, Geologiche e Ambientali – Sezione di Scienze della Terra, Corso Italia 57, I-95129 Catania, Italy; 2 Istituto Nazionale di Geofisica e Vulcanologia (INGV) - Sezione di Catania, Osservatorio Etneo, Piazza Roma 2, 95125 Catania, Italy; 3 Consiglio Nazionale delle Ricerche (CNR), Istituto di Geoscienze e Georisorse (IGG) – Sezione di Pavia, Via A. Ferrata 1, I-27100 Pavia, Italy

## Abstract

Lithium gradients in plagioclase are capable of recording extremely short-lived processes associated with gas loss from magmas prior to extrusion at the surface. We present SIMS profiles of the ^7^Li/^30^Si ion ratio in plagioclase crystals from products of the paroxysmal sequence that occurred in the period 2011–2013 at Mt. Etna (Italy) in an attempt to constrain the final ascent and degassing processes leading to these powerful eruptions involving basic magma. The observed Li concentrations reflect cycles of Li addition to the melt through gas flushing, and a syn-eruptive stage of magma degassing driven by decompression that finally produce significant Li depletion from the melt. Modeling the decreases in Li concentration in plagioclase by diffusion allowed determination of magma ascent timescales that are on the order of minutes or less. Knowledge of the storage depth beneath the volcano has led to the quantification of a mean magma ascent velocity of ~43 m/s for paroxysmal eruptions at Etna. The importance of these results relies on the application of methods, recently used exclusively for closed-system volcanoes producing violent eruptions, to open-conduit systems that have generally quiet eruptive periods of activity sometimes interrupted by sudden re-awakening and the production of anomalously energetic eruptions.

## Introduction

Modes and timescales of magma ascent and degassing in the shallow portion of a volcano plumbing system (upper few kilometers) are crucial to understanding the dynamics of an eruption. The rate at which the magma rises to the surface controls the capability of magmas to lose the gas phase that in turn influences the effusive or explosive behavior of an eruption^1,2^. Syn-eruptive ascent and decompression rates of magmas in explosive eruptions have been inferred through experimental simulation^3,4^ or from textural characteristics of the emitted products^5–8^. However, direct measurements of ascent velocity during volcanic eruptions are not routinely constrained through *in-situ* analyses on minerals and melts. One obstacle to such kind of studies is that the ascent velocities of gas-rich magmas and their vesiculation upon eruption may be extremely fast (hours, minutes or even less), which makes them difficult to assess with standard geospeedometers, such as diffusion of elements in various minerals (Fe-Mg in olivine, Mg, Sr, Ba in plagioclase). Recent studies invoked the diffusion of volatile species between melt and bubbles as a new method to quantify magma ascent and decompression rates^9–11^. Other methods involved the investigation of intra-crystalline diffusion of light elements, such as Li, Be and B that are markers of magma degassing^12,13^. In particular, concentration gradients of Li in plagioclase crystals have proved to be useful for the identification and quantification of fast processes of magma ascent and degassing that occur shortly before, and even during eruptions^12,14–17^. The increasing interest in studying Li concentrations and its diffusive processes is based on the observation of the extremely mobile behaviour of Li, which easily migrates into the fluid phase at low pressure^18^ with rates faster than those of water^19^. Moreover, the fluid-melt partition coefficient of Li increases with decreasing pressure^18^. The rates of Li diffusion in plagioclase are 10^−10^-10^−12^ m^2^ s^−1^ for a temperature range of 200–850 °C, i.e. orders of magnitude faster than NaSi-CaAl interdiffusion^20–22^. This means that Li diffusion is sufficiently rapid to constrain short-lived magmatic processes, because any Li concentration gradient in plagioclase would be rapidly homogenised through diffusion to the equilibrium profile, unless arrested by sudden cooling of magma after emission^12^. Lithium has been successfully used for time constraints of syn-eruptive degassing in volcanic systems erupting products of various compositions (basalts to rhyolite), whose degassing occurs primarily under closed-system conditions (e.g., Taupo, New Zealand; Volcàn de Fuego, Guatemala; Nea Kameni Santorini, Greece; Mt. St. Helens, USA).

We report here the results of Secondary Ion Mass Spectrometry (SIMS) investigations of Li in plagioclase crystals found in agglutinates and rheomorphic lava flows produced by Mt. Etna volcano during the violent paroxysms that occurred during the 2011–2013 AD period at the New South East Crater, and which were sampled and quenched immediately after the emplacement. The paroxysmal eruptions (44 episodes throughout 2011–2013 AD) considered in this study have been similar to each other regarding the composition of the erupted products and eruptive behaviour^23^. During each paroxysmal eruption, the evolution of the volcanic phenomena was characterized by varying durations of an early weak- to mildly-Strombolian activity, shifting later to violent Strombolian activity and vigorous lava fountaining (~1-km-high above the crater edge) that produced eruptive columns up to several km high^23,24^. The novelty of the present study arises from the application of Li diffusion modeling to unraveling the timescales of syn-eruptive decompression-driven degassing at Mt. Etna, which is generally acknowledged to degas through open-system conditions, but is also able to produce extremely energetic eruptions.

## Analytical Procedures

Thin sections representative of agglutinates and rheomorphic lavas from various paroxysmal events occurred at Mt. Etna in the period 2011–2013 have been selected and prepared for the ion microprobe. SIMS investigations were performed on selected plagioclase crystals and on the matrix glass immediately adjacent to the selected crystals. Among plagioclase crystals preserved in lava rocks from the recent eruptive activity at Mt. Etna volcano, seven crystals were chosen because they are totally oscillatory-zoned plagioclases, indicating crystal growth close to equilibrium. The thin sections were polished, washed in an ultrasonic tank, and Pt-coated prior to SIMS analysis conducted using the IMS 4 f Cameca ion microprobe installed at CNR-IGG (Pavia). We used a 5 nA ^16^O^–^, 6–8 µm Ø primary beam to sputter secondary ions from micro-areas chosen along transects within the crystals and in the adjacent matrix glass. The analytical set up was as follows: −12.5 kV accelerating voltage, 25-µm secondary-ion imaged field, 400-µm contrast aperture, 1800-µm field aperture, and 900 (M/ΔM) mass resolving power. The energy filtering technique of secondary ions (range: 75–125 eV) was adopted to reduce matrix effects and improve analytical precision^25,26^. ^7^Li^+^ and ^30^Si^+^ were monitored over 5 analytical cycles with 8 and 4 sec acquisition time each - for a total time of 40 and 20 sec, respectively - after a 450-sec waiting time to obtain steady-state sputtering conditions. NIST-SRM-610, −612 and −614 international standards were used as calibration samples in order to convert the ion signal for Li into Li concentrations (ppm, wt.%)^27,28^. The analytical reproducibility of the ^7^Li^+^/^30^Si^+^ ratios resulted to be ±1% (1σ) for NIST-SRM-610 over a day. The experimental data for all the crystals were collected in four, one-day analytical sessions. After SIMS investigations, the thin sections were then smoothly re-polished, and analysed at the Tescan Vega-LMU SEM (equipped with an EDAX Neptune XM4-60 micro-analyzer and coupled with an EDAX EDS and WDS LEXS spectrometer, calibrated for light elements), at Dipartimento di Scienze Biologiche, Geologiche e Ambientali (University of Catania) to determine major element composition and the proper anorthite content relative to each SIMS spot. Operating conditions were the following: 20 kV accelerating voltage, 2 nA beam current and ~2 μm Ø focused electron beam. Repeated analyses on internationally certified minerals and internal glass standards ensured precision for all the collected elements on the order of 3–5%.

The SEM-EDS/WDS SiO_2_ (wt.%) values at each spot were finally adopted in the SIMS quantification of Li. Moreover, in order to further reduce the residual matrix effects and take into account the different SiO_2_ (wt.%) values among the plagioclase crystals and the NIST glasses (~50 wt.% SiO_2_ vs. 72.2 wt.% SiO_2_ in the NIST-series glasses), we applied a correction to the relative-to-Si ion yield for Li, i.e., I(Li)/I(Si) / Li(at)/Si(at) [where I(Li) and I(Si) represent the ionic signals for Li and Si, and Li(at) and Si(at), their respective atomic concentrations]^25^. The accuracy of the final SIMS data for Li concentration is quoted better than 10% rel. The anorthite composition determined at each SIMS spot was used to correct the measured ^7^Li/^30^Si ratios for the variation in silica. Specifically, the ^30^Si count rate was converted into ^30^Si_i_, which is proportional to the amount of plagioclase sputtered during the analysis. This conversion follows the equation:

1 $${}^{30}S{i}_{i}={}^{30}Si\{(4/3)+2{X}_{An}/3\}$$

where the ^30^Si_i_ value accounts for the number of tetrahedral sites sputtered during SIMS bombardment, assuming constant ionization efficiency for Si^17^. Therefore, the ^7^Li/^30^Si_i_ signal ratio represents a value proportional to the molar Li content within the plagioclase, i.e. proportional to the number of Li atoms for the tetrahedral site^17^.

## Results and Discussion

**Figure 1:**
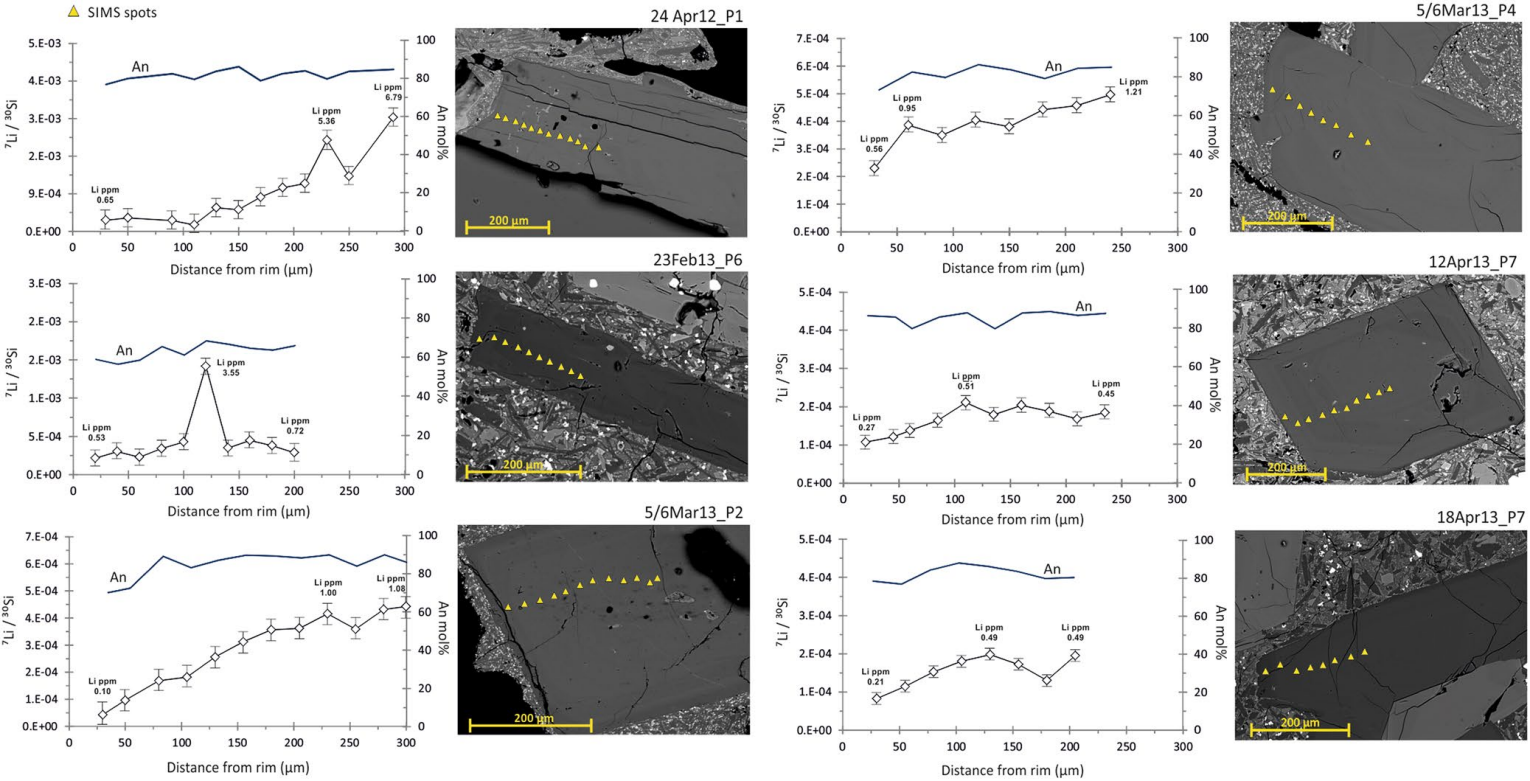
Ion microprobe analysis spots for ^7^Li/^30^Si (An-corrected) versus distance from crystals edge plotted together with the An mol% content (SEM-EDS/WDS analysis) for plagioclase crystals in this study. Error bars are the standard deviation of the mean of measurements (±1σ).

Crystals used in this study are An_72-90_ plagioclases with sizes between ~500 and 950 μm. Only plagioclase 23Feb13_P6 represents an exception, as it is more Ab-rich than the other crystals (An_56-68_) meaning that it may have experienced crystallization in a more differentiated melt. In general, anorthite variation throughout the core-to-rim profile is narrow (i.e. An oscillation range in the order of 5–10 mol%), and can show either slight increases or decreases at the outermost edge. In all crystals, Li concentration was measured as variations of the ^7^Li/^30^Si ion ratios along 200–300 μm-long transects that follow the apparent plagioclase c-axis. ^7^Li^+^/^30^Si^+^ values range from 2.7 × 10^−3^ to 0.4 × 10^−4^, corresponding to absolute Li concentrations between 6.8 ppm and 0.1 ppm (Fig. 1). It is worth noting that for this study we did not measure ^6^Li/^7^Li ratios, in spite of the potential for this ratio to be a good indicator for diffusion^29,30^, because the counting rates at the mass of ^6^Li were insufficient to produce robust counting statistics. Examinations of the Li SIMS profiles have shown overall trends of decreasing concentrations from the crystal interior toward the edge, without specific correlations to the An concentration profile of each crystal. Throughout the analysed spatial ranges, we also recognized localized spikes in the Li content that shifts to higher values at rather regular intervals within the plagioclase (Fig. 1). SIMS measurements of the ^7^Li/^30^Si ratio in the matrix glass immediately adjacent to the analysed plagioclase crystals provide evidence of a significant variability in the Li concentration of the plagioclase host melt. Glass data indicate ^7^Li/^30^Si ion ratios of one-two orders of magnitude higher compared to values in plagioclase, corresponding to actual Li concentrations between 0.5 and 29 ppm.

Decreasing concentrations of Li toward the edge of plagioclase crystals have been interpreted as superimposition of the effect of mineral-melt partitioning and loss of Li from the melt due to preferential transport of Li into the gas phase during the eruption^14–16^. Former experimental studies of plagioclase-melt partition coefficients demonstrate that Li is moderately incompatible in An_39-77_ plagioclase (K_D_ = 0.15-0.7) and that crystal-melt partition coefficient does not vary systematically with changing X_An_ at constant temperature^31–33^. However, these experiments highlighted that the melt composition may play a role in controlling the partition coefficient of Li between crystals and melt. Indeed, more recent experiments proved that the partitioning of Li into plagioclase is a function of the An contents over the composition range An_60_-An_89_, decreasing of a factor of ~3.5 from An_60_ to An_80_^34^. Anorthite variation of the Etnean crystals is, however, rather limited (ΔAn is only 5–10 mol%) and does not correlate with the decreasing Li profile. This implies that changes in Li partitioning behavior are unlikely to fully explain the observed depletion of Li towards the crystal rims. Moreover, the relatively homogeneous melts reported for the recent eruptive products of Etna^23^ suggest that changes in the plagioclase/melt partition coefficient for Li due to compositional changes of the melt would be small, and insufficient to explain the observed concentration profiles. Unless the plagioclase crystals were mixed with a Li-poor magma during its final stages of growth, the general rim-wards decline of Li concentrations can be only interpreted as a consequence of diffusive Li disequilibrium related to volatile exsolution during magma ascent. Despite magma ascent and degassing paths for past eruptions of Mt. Etna being well constrained by several melt inclusion studies^35–40^, the existence of a sufficiently low-Li magma beneath Etna that would explain the observed concentration profiles is not supported by measurements of Li in coexisting melt inclusions. The few data available indicate that Li contents in Etnean melt inclusions vary from 3.4–10.2 ppm for a wide range of eruptions that occurred in historic and recent times^41–44^. In particular, melt inclusions entrapped in crystals of recent products at pressure lower than 300 MPa (<7–10 km b.s.l.)^44^ have Li contents of 7.8–9.9 ppm, much higher than those measured in our plagioclase crystals. Given the incompatibility of Li in all mineralogical phases, the amount of Li should progressively increase in the residual melt through fractional crystallization. In this regard, the high Li concentration of the matrix glass confirms the absence of Li-poor melts in which crystals may have been stored for a given time, sufficient to produce the observed Li concentration gradients (see ESM1 in the Supplementary Information for compositions of matrix glasses). Excluding a major role played by Li-melt partitioning or the entrainment into a Li-poor magma volume, the observed decreases in Li in plagioclase crystals must reflect, therefore, rapid decreases in melt Li presumably due to ascent-driven degassing. This inference is robust considering that Li gradients have been measured only in oscillatory-zoned crystals, which are assumed to crystallize under rather unperturbed chemical and physical conditions. This should remove the potential contribution deriving from the superimposition of other pre-eruptive magmatic processes on the plagioclase Li concentration before the final stage of degassing. Our data also support the idea that depletion of Li from the melt due to volatile exsolution was an incomplete process, as the groundmass glass compositions are far too high in Li to be in equilibrium with the plagioclase Li contents.

The occurrence of localized Li spikes that are superimposed on the main trends of decreasing Li within the plagioclase requires an increase in Li abundance in the host melt. Compositional trends characterized by increasing Li toward the margin of plagioclase were previously observed during the investigation of products of the ~27 ka Oruanui eruption (Taupo, New Zealand), and interpreted as a consequence of changing Li partitioning behaviour due to extensive release of Cl from the system during decompression^17^. The discharge of a significant amount of Cl from a melt at subcritical fluid conditions was found to affect the partitioning of Li that forms LiOH hydroxides. This process would make Li far less compatible in the melt, increasing its chemical activity, and thus providing the driving force for Li diffusion back into plagioclase crystals^17^. Etnean trachybasalts are acknowledged to be volatile-rich, and the Cl concentration in the melt is significant, ranging between 0.05 and 0.46 wt.%^37,38,40^. However, the preferential partitioning of Li into the melt driven by extensive loss of Cl cannot account for the development of localized Li peaks in these Etnean products. Indeed, the exsolution of a Cl-bearing volatile phase in Etnean magmas begins at ~100 MPa^37,38,40^ and becomes more prominent due to ascent-driven decompression. Therefore, if we assume that Cl exsolution effectively changes the Li partitioning, enhancing the preferential partitioning of Li back into the melt, this mechanism would produce a continuous trend of Li increase toward the plagioclase edge^17^, which is clearly in contrast with what we observed. Moreover, based on compositional observations on plagioclase crystals erupted during 2011 and 2013 at Mt. Etna, magmatic recharge and mixing events cannot be responsible for changes in melt Li contents, as fluctuations in minor elements such as Fe, Mg and/or Ti do not correlate with Li spikes^24^. We believe that the observed short-length scale Li peaks within plagioclase crystals likely reflect transient dynamic processes associated with gas migration from deep portions of the Etna transport system. Published data on primitive glass inclusions in olivines confirm that the volatile-rich magmas of Mt. Etna can exsolve a H_2_O-CO_2_ gas phase at pressure higher than 250 MPa^36–40^, which continuously flush the overlying magma reservoirs stored at lower pressure^45^. These processes are thought responsible for the high explosivity of Etna eruptions in the past or during the last decades, as they act as a recurrent trigger mechanism for high-energy explosive events^40,46–48^. In accordance with these observations, Li-bearing gases released from the deep levels of the volcano transport system may systematically rise up and accumulate in shallow reservoirs, where they partially re-equilibrate with the melt during storage, and hence serve to increase the Li content of the melt. Under these conditions, the longer a magma body is stored at low pressures, the greater the addition of Li to the melt^14,49^. In spite of a lack of published data regarding Li distribution in melt inclusions to confirm the ascent of Li-rich gas phases at Mt. Etna, the occurrence of multiple peaks at higher Li concentration is likewise best explained via repeated cycles of gas flushing in shallow magma reservoirs, which are systematically recorded by plagioclase during its growth. The presence of short length scale Li spikes reflects heterogeneous compositions acquired by the crystal as a consequence of flushing before the final stage of degassing. Given that diffusion tends to erase any concentration gradient, this initial heterogeneity is also affected by diffusive relaxation. Preservation of Li spikes is therefore possible if diffusive relaxation due to final degassing is not complete, so that the crystal preserves a record of the initial heterogeneity that is superimposed on the main trend of Li decreasing toward the rim.

**Figure 2:**
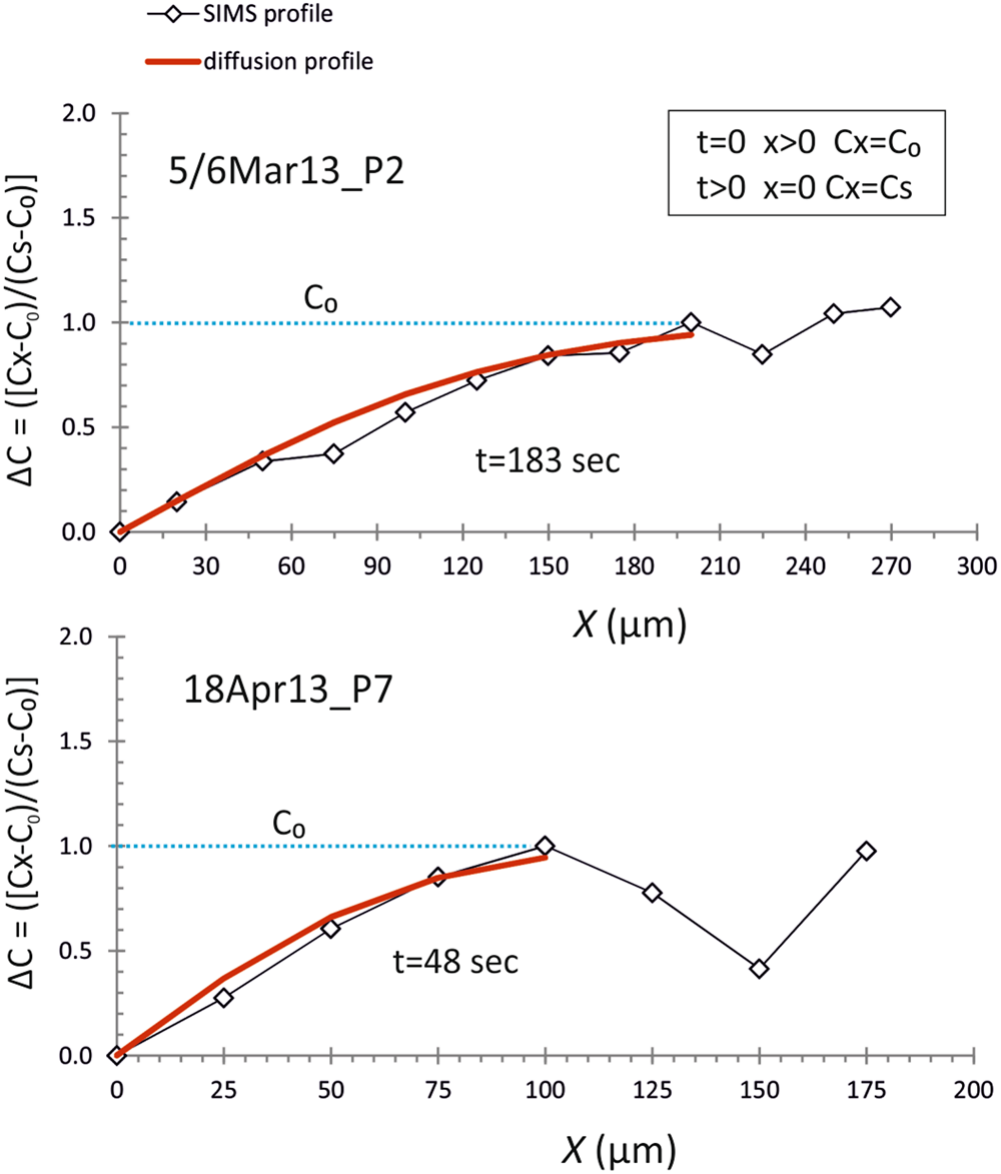
Examples of one-dimensional diffusion modeling application for Li profiles in Etnean plagioclase crystals. Light blue dashed lines indicate the initial concentration profile of the crystal (C_0_), whereas the red lines represent the best-fit diffusion curves. ΔC (*y*-axes) refers to the normalized compositional contrast for Li in plagioclase. (see ESM2 in the Supplementary Information for all the modeled crystals).

All the previous inferences support the development of plagioclase compositional profiles as expression of cycling addition and final diffusive relaxation of Li, which is a consequence of chemical disequilibrium between plagioclase and Li-depleted melt due to degassing. Modelling the diffusion of lithium in plagioclase provides us the opportunity to assess the timing of syn-eruptive decompression-driven degassing of magmas associated with the recent paroxysmal activity at Mt. Etna. We modelled each lithium profile using a one-dimensional model of diffusion in a semi-infinite solid, assuming uniform initial concentration in the crystal and constant concentration at the diffusion surface as initial and boundary conditions for the error function^50^ (Fig. 2). The adopted approach allowed the derivation of analytical solutions for the following diffusion equation:

2 $$[({C}_{x}-{C}_{0})/(Cs-{C}_{0})]=1-erf[x/2{(Dt)}^{0.5}]$$

where *C*_x_ represents the composition at the distance *x* from the surface, *D* is the diffusion coefficient for Li in plagioclase, and *t* is the time elapsed since diffusion begins. The diffusive evolution through time of Li was modelled with a composition-independent diffusion coefficient *D* of 3.04×10^−11^ m^2^/s, given the lack of correlation between the Li diffusion coefficient and major element concentrations in plagioclase^20^. The concentration independence of the diffusion coefficient allowed using an analytical solution for the modelling, and thus we do not need to rely on more complex numerical solutions^51,52^. Coefficients were calculated at 1080 °C by using the Arrhenius parameters (i.e., the pre-exponential factor *D*_0_, and the activation energy Q) for Li diffusivity in plagioclase of anorthitic composition^20^. The choice of temperature is in accordance with the eruptive temperatures obtained at Mt. Etna by geothermometers calibrated for Etnean basalts^53^. It is worth noting that Li diffusion coefficients in plagioclase have been experimentally calibrated only for the temperature range 200–850 °C^20^. However, extrapolation of the coefficient for higher temperatures has been made possible due to the linear correlation between the log*D*_Li_ and temperature^20^. Diffusion modelling calculations yielded average timescales of syn-eruptive magma ascent and degassing for the recent paroxysmal eruptions at Mt. Etna of 84 ± 10 seconds (Table 1). Our model led to the longest timescales for magmas feeding the eruption of March 5–6, 2013 (170 ± 21 to 183 ± 23 seconds), whereas significantly shorter timescales were estimated for the February 23, 2013 eruption (10 ± 1 seconds). Uncertainties on these timescales were calculated by propagating the error in the determination of diffusion coefficient *D*, through the relationship *t* = *x* ^2^/4*D*, where *x* ^2^ represents the diffusion length (in µm) within the profile, and *t* is the time^54^. The uncertainty on *D* was calculated using standard procedures for error propagation in the Arrhenius expression, also taking into account uncertainties in the experimental determination of *D*_0_ and Q for Li diffusivity in anorthite^20^. Based on the error propagation analysis, the uncertainties on the calculated timescales are mainly governed by temperature changes, which largely affect *D*, in a way that a temperature of 1080 ± 10 °C produces a propagating uncertainty in timescales from ±0.2 to ±20 seconds.

**Table 1:** Time estimates of the Li diffusion from concentration profiles in plagioclase crystals of recent eruptions at Mt. Etna. All timescales calculated for diffusivities *D* of 3.04 × 10^−11^ m^2^/s at 1080 °C.

Crystal	Eruption date	Diffusion width (µm)	Time (seconds)	2σ
24Apr12_P1	April 24, 2012	210	60	±7
24Apr12_P3	April 24, 2012	150	82	±10
23Feb13_P6	February 23, 2013	100	10	±1
5/6Mar13_P2	March 5-6, 2013	200	183	±23
5/6Mar13_P4	March 5-6, 2013	210	170	±21
12Apr13_P7	April 12, 2013	90	38	±5
18Apr13_P7	April 18, 2013	100	48	±6

These time estimates offer us the opportunity to determine the final rates of magma degassing and ascent associated with paroxysmal eruptions at Mt. Etna. Determinations of the ascent rate upon eruption require, however, some constraints on the pressure at which Li disequilibrium begins and, therefore, the pressure at which the magma was stored before eruption. In this regard, the compositional and textural characteristics of plagioclase crystals from the post-2011 activity at Mt. Etna highlighted common late-stage histories of crystallization in a shallow storage zone^24,55,56^. This magma reservoir was inferred to be located below the summit area at 1.5–2 km a.s.l., corresponding to a pressure of ~40 MPa^57,58^. This indicates that just prior to the eruption, magmas were likely stored at an average depth of ~1.6 km beneath the volcano summit, and later underwent instantaneous decompression through the conduit. Assuming that the Li diffusion begins at this depth, our timescales imply average rates of final magma ascent on the order of 43 m/s for the considered highly explosive episodes at Mt. Etna. These ascent velocities for Etnean magmas are in the same range of rates estimated through diffusion modelling for a number of extremely powerful eruptions worldwide (4–64 m/s). The crucial findings of this study demonstrate that re-activation of the magmatic systems at open-conduit, basaltic volcanoes can occur with exceptionally fast timescales, which are on the whole comparable with those for syn-eruptive degassing derived for other closed-system volcanoes erupting on Earth, and is followed by the generation of highly energetic eruptions.

## Electronic supplementary material

Supplementary Material 1

Supplementary Material 2
